# Supplementary material for: HIVprotI: an integrated web based platform for prediction and design of HIV proteins inhibitors
Source: J Cheminform. 2018 Mar 9;10:12. doi: 10.1186/s13321-018-0266-y (PMC5845081; doi:10.1186/s13321-018-0266-y)
Supplement: Supplementary file 2 — Additional file 2. Source code of HIVProtI web server. [file 13321_2018_266_MOESM2_ESM.zip › HIVprotI_Source-code/hivprotI/weka-3-6-6/doc/weka/core/converters/ConverterUtils.html]

ConverterUtils


---


|  |  |  |
| --- | --- | --- |
| **Overview** \| **Package** \| **Class** \| **Tree** \| **Deprecated** \| **Index** \| **Help** \| **Weka's home** | |  |
| **PREV CLASS**   **NEXT CLASS** | **FRAMES**    **NO FRAMES**     **All Classes** |  |
| SUMMARY: NESTED \| FIELD \| CONSTR \| METHOD | DETAIL: FIELD \| CONSTR \| METHOD |  |


---


## weka.core.converters Class ConverterUtils

```
java.lang.Object
  weka.core.converters.ConverterUtils
```

**All Implemented Interfaces:**: java.io.Serializable, RevisionHandler

---

``` public class ConverterUtils extends java.lang.Object implements java.io.Serializable, RevisionHandler ```

Utility routines for the converter package.

**Version:**
:   $Revision: 6416 $

**Author:**
:   Mark Hall (mhall@cs.waikato.ac.nz), FracPete (fracpete at waikato dot ac dot nz)

**See Also:**: `Serializable`, Serialized Form

---

| **Nested Class Summary** | |
| --- | --- |
| `static class` | `ConverterUtils.DataSink`             Helper class for saving data to files. |
| `static class` | `ConverterUtils.DataSource`             Helper class for loading data from files and URLs. |


| **Field Summary** | |
| --- | --- |
| `static java.lang.String` | `CORE_FILE_LOADERS`             the core loaders - hardcoded list necessary for RMI/Remote Experiments (comma-separated list). |
| `static java.lang.String` | `CORE_FILE_SAVERS`             the core savers - hardcoded list necessary for RMI/Remote Experiments (comma-separated list). |


**Constructor Summary**

`ConverterUtils()`


| **Method Summary** | |
| --- | --- |
| `static void` | `errms(java.io.StreamTokenizer tokenizer, java.lang.String theMsg)`             Throws error message with line number and last token read. |
| `static java.util.Vector<java.lang.String>` | `getFileLoaders()`             returns a vector with the classnames of all the file loaders. |
| `static java.util.Vector<java.lang.String>` | `getFileSavers()`             returns a vector with the classnames of all the file savers. |
| `static void` | `getFirstToken(java.io.StreamTokenizer tokenizer)`             Gets token, skipping empty lines. |
| `static AbstractFileLoader` | `getLoaderForExtension(java.lang.String extension)`             tries to determine the loader to use for this kind of extension, returns null if none can be found. |
| `static AbstractFileLoader` | `getLoaderForFile(java.io.File file)`             tries to determine the loader to use for this kind of file, returns null if none can be found. |
| `static AbstractFileLoader` | `getLoaderForFile(java.lang.String filename)`             tries to determine the loader to use for this kind of file, returns null if none can be found. |
| `java.lang.String` | `getRevision()`             Returns the revision string. |
| `static AbstractFileSaver` | `getSaverForExtension(java.lang.String extension)`             tries to determine the saver to use for this kind of extension, returns null if none can be found. |
| `static AbstractFileSaver` | `getSaverForFile(java.io.File file)`             tries to determine the saver to use for this kind of file, returns null if none can be found. |
| `static AbstractFileSaver` | `getSaverForFile(java.lang.String filename)`             tries to determine the saver to use for this kind of file, returns null if none can be found. |
| `static void` | `getToken(java.io.StreamTokenizer tokenizer)`             Gets token. |
| `static java.util.Vector<java.lang.String>` | `getURLFileLoaders()`             returns a vector with the classnames of all the URL file loaders. |
| `static AbstractFileLoader` | `getURLLoaderForExtension(java.lang.String extension)`             tries to determine the URL loader to use for this kind of extension, returns null if none can be found. |
| `static AbstractFileLoader` | `getURLLoaderForFile(java.io.File file)`             tries to determine the URL loader to use for this kind of file, returns null if none can be found. |
| `static AbstractFileLoader` | `getURLLoaderForFile(java.lang.String filename)`             tries to determine the URL loader to use for this kind of file, returns null if none can be found. |
| `static boolean` | `isCoreFileLoader(java.lang.String classname)`             checks whether the given class is one of the hardcoded core file loaders. |
| `static boolean` | `isCoreFileSaver(java.lang.String classname)`             checks whether the given class is one of the hardcoded core file savers. |

**Methods inherited from class java.lang.Object**

`equals, getClass, hashCode, notify, notifyAll, toString, wait, wait, wait`

**Field Detail**

### CORE\_FILE\_LOADERS

```
public static final java.lang.String CORE_FILE_LOADERS
```

:   the core loaders - hardcoded list necessary for RMI/Remote Experiments
    (comma-separated list).

---


### CORE\_FILE\_SAVERS

```
public static final java.lang.String CORE_FILE_SAVERS
```

:   the core savers - hardcoded list necessary for RMI/Remote Experiments
    (comma-separated list).


**Constructor Detail**

### ConverterUtils

```
public ConverterUtils()
```


**Method Detail**

### getFirstToken

```
public static void getFirstToken(java.io.StreamTokenizer tokenizer)
                          throws java.io.IOException
```

:   Gets token, skipping empty lines.

    :   **Parameters:**: `tokenizer` - the stream tokenizer **Throws:**: `java.io.IOException` - if reading the next token fails

---


### getToken

```
public static void getToken(java.io.StreamTokenizer tokenizer)
                     throws java.io.IOException
```

:   Gets token.

    :   **Parameters:**: `tokenizer` - the stream tokenizer **Throws:**: `java.io.IOException` - if reading the next token fails

---


### errms

```
public static void errms(java.io.StreamTokenizer tokenizer,
                         java.lang.String theMsg)
                  throws java.io.IOException
```

:   Throws error message with line number and last token read.

    :   **Parameters:**: `theMsg` - the error message to be thrown: `tokenizer` - the stream tokenizer **Throws:**: `java.io.IOException` - containing the error message

---


### isCoreFileLoader

```
public static boolean isCoreFileLoader(java.lang.String classname)
```

:   checks whether the given class is one of the hardcoded core file loaders.

    :   **Parameters:**: `classname` - the class to check **Returns:**: true if the class is one of the core loaders **See Also:**: `CORE_FILE_LOADERS`

---


### getFileLoaders

```
public static java.util.Vector<java.lang.String> getFileLoaders()
```

:   returns a vector with the classnames of all the file loaders.

    :   **Returns:**: the classnames of the loaders

---


### getLoaderForFile

```
public static AbstractFileLoader getLoaderForFile(java.lang.String filename)
```

:   tries to determine the loader to use for this kind of file, returns
    null if none can be found.

    :   **Parameters:**: `filename` - the file to return a converter for **Returns:**: the converter if one was found, null otherwise

---


### getLoaderForFile

```
public static AbstractFileLoader getLoaderForFile(java.io.File file)
```

:   tries to determine the loader to use for this kind of file, returns
    null if none can be found.

    :   **Parameters:**: `file` - the file to return a converter for **Returns:**: the converter if one was found, null otherwise

---


### getLoaderForExtension

```
public static AbstractFileLoader getLoaderForExtension(java.lang.String extension)
```

:   tries to determine the loader to use for this kind of extension, returns
    null if none can be found.

    :   **Parameters:**: `extension` - the file extension to return a converter for **Returns:**: the converter if one was found, null otherwise

---


### getURLFileLoaders

```
public static java.util.Vector<java.lang.String> getURLFileLoaders()
```

:   returns a vector with the classnames of all the URL file loaders.

    :   **Returns:**: the classnames of the loaders

---


### getURLLoaderForFile

```
public static AbstractFileLoader getURLLoaderForFile(java.lang.String filename)
```

:   tries to determine the URL loader to use for this kind of file, returns
    null if none can be found.

    :   **Parameters:**: `filename` - the file to return a URL converter for **Returns:**: the converter if one was found, null otherwise

---


### getURLLoaderForFile

```
public static AbstractFileLoader getURLLoaderForFile(java.io.File file)
```

:   tries to determine the URL loader to use for this kind of file, returns
    null if none can be found.

    :   **Parameters:**: `file` - the file to return a URL converter for **Returns:**: the converter if one was found, null otherwise

---


### getURLLoaderForExtension

```
public static AbstractFileLoader getURLLoaderForExtension(java.lang.String extension)
```

:   tries to determine the URL loader to use for this kind of extension, returns
    null if none can be found.

    :   **Parameters:**: `extension` - the file extension to return a URL converter for **Returns:**: the converter if one was found, null otherwise

---


### isCoreFileSaver

```
public static boolean isCoreFileSaver(java.lang.String classname)
```

:   checks whether the given class is one of the hardcoded core file savers.

    :   **Parameters:**: `classname` - the class to check **Returns:**: true if the class is one of the core savers **See Also:**: `CORE_FILE_SAVERS`

---


### getFileSavers

```
public static java.util.Vector<java.lang.String> getFileSavers()
```

:   returns a vector with the classnames of all the file savers.

    :   **Returns:**: the classnames of the savers

---


### getSaverForFile

```
public static AbstractFileSaver getSaverForFile(java.lang.String filename)
```

:   tries to determine the saver to use for this kind of file, returns
    null if none can be found.

    :   **Parameters:**: `filename` - the file to return a converter for **Returns:**: the converter if one was found, null otherwise

---


### getSaverForFile

```
public static AbstractFileSaver getSaverForFile(java.io.File file)
```

:   tries to determine the saver to use for this kind of file, returns
    null if none can be found.

    :   **Parameters:**: `file` - the file to return a converter for **Returns:**: the converter if one was found, null otherwise

---


### getSaverForExtension

```
public static AbstractFileSaver getSaverForExtension(java.lang.String extension)
```

:   tries to determine the saver to use for this kind of extension, returns
    null if none can be found.

    :   **Parameters:**: `extension` - the file extension to return a converter for **Returns:**: the converter if one was found, null otherwise

---


### getRevision

```
public java.lang.String getRevision()
```

:   Returns the revision string.

    :   **Specified by:**: `getRevision` in interface `RevisionHandler`
    :   **Returns:**: the revision


---


|  |  |  |
| --- | --- | --- |
| **Overview** \| **Package** \| **Class** \| **Tree** \| **Deprecated** \| **Index** \| **Help** \| **Weka's home** | |  |
| **PREV CLASS**   **NEXT CLASS** | **FRAMES**    **NO FRAMES**     **All Classes** |  |
| SUMMARY: NESTED \| FIELD \| CONSTR \| METHOD | DETAIL: FIELD \| CONSTR \| METHOD |  |


---
